# Checklist: A Useful and Safe Tool for the Initiation of Care for Eutocical Vaginal Delivery

**DOI:** 10.3390/ijerph192013409

**Published:** 2022-10-17

**Authors:** María Fernández Muñoz, Zurine Raquel Reyes Angullo, Pilar Pintado Recarte, Lucia Consuelo Soto, Javier Ruiz Labarta, Ignacio Cueto Hernández, Miguel A. Ortega, Juan A. De Leon-Luis

**Affiliations:** 1 Department of Public and Maternal and Child Health, School of Medicine, Complutense University of Madrid, 28040 Madrid, Spain; 2 Department of Obstetrics and Gynecology, University Hospital Gregorio Marañón, 28009 Madrid, Spain; 3 Health Research Institute Gregorio Marañón, Consortium for Biomedical Research in Epidemiology and Public Health (CIBERESP), 28009 Madrid, Spain; 4 Maternal and Infant Research Investigation Unit, Alonso Family Foundation (UDIMIFFA), 28009 Madrid, Spain; 5 Department of Medicine and Medical Specialties, Faculty of Medicine and Health Sciences, University of Alcalá, 28801 Madrid, Spain; 6 Ramón y Cajal Institute of Healthcare Research (IRYCIS), 28034 Madrid, Spain

**Keywords:** eutocic vaginal delivery, simulation, checklist, learning, obstetrics

## Abstract

The aim of this study is to present and analyze the vaginal delivery checklist as a safe and useful learning tool for first-year residents in the gynecology and obstetrics and midwifery specialties at different hospitals and to analyze the items that comprise it and the progress of the residents during the first 30 normal deliveries attended in a supervised manner. We present a descriptive, observational and prospective study in which 18 participants from different hospitals in Spain completed a checklist of skills in the first 30 births attended autonomously after learning its use in a simulation workshop. We collected a total of 329 of 330 checklists completed by 11 participants. In addition, the mean responses for each item contained on the checklist and the mean global progression of the participants were subsequently analyzed, detecting statistically significant differences using Student’s t-test for paired data. During the data collection period between September 2020 and August 2021, a total of 329 checklists were analyzed. The global average of positive responses for the 30 questions contained on the checklist was 25.36 out of 30 points. The fulfillment of the different items was variable, with the lowest fulfillment of 2.18 points, although 73% of the items obtained a score greater than 25. A statistically significant difference in the mean compliance of the first five childbirths compared to the last five deliveries using the proposed checklist was obtained, with *p* < 0.01. In conclusion, knowing the importance of using a checklist to anticipate risk situations and reduce the number of adverse perinatal outcomes, we can say that, for first-year residents in gynecology and obstetrics attending a clinical simulation workshop in eutocic vaginal delivery, it may be interesting to have a tool, such as the checklist proposed in this study, that facilitates the learning process and the suitable progress of the participants.

## 1. Introduction

The implementation of good obstetric practices and the prevention of maternal and neonatal morbidity and mortality are the goals of obstetric care. In this way, the training and qualification of professionals for adequate assistance to eutocic vaginal delivery (EVD) are central points in improving the care provided in our hospitals [1]. One of the ways to improve the training of professionals involved in obstetric clinical interventions is through the establishment of a common protocol and checklist (CL) that is applicable, safe and reproducible [2].

Eutocic vaginal delivery (EVD) is a stressful situation for professionals with little experience due to the difficulty of access to learning during such a sensitive and intimate procedure for patients.

Multiple tools have been defined for teaching EVD, but the most effective and safest method for its application in clinical practice has not been demonstrated [3]. In addition, the number of deliveries, both simulated and real, required to acquire adequate training is unknown [4].

It is necessary to analyze a useful and safe tool that provides knowledge of the learning curve, facilitating access for future first-year residents in gynecology and obstetrics (RGyO), doctors and midwives to a controlled and reproducible clinical practice.

For this reason, we propose a CL as a fundamental tool to improve clinical care for EVD. The CL contains essential items to provide safe and controlled EVD care in our environment, based on the recommendations of the main health authorities [5,6].

In our study, the RGyO were trained in the completion of CLs after attending a clinical simulation workshop in eutocic vaginal delivery (SW-EVD). The training and qualification of the different professionals who attend the birth process are essential in reducing both maternal and fetal mortality and morbidity [7]. Although the quality of the studies is heterogeneous with varied indicators, the evidence has shown that simulation-based teaching is effective and leads to better and more lasting results than traditional teaching, both in routine situations and in times of emergency. This outcome is demonstrated in part by the increased security that participants achieve in this type of learning, finding themselves in a risk-free environment that allows them to be placed in different complex situations without harming third parties [8].

The objective of this study was to evaluate the degree of compliance with each item that constitutes the CL tool after having attended between 25 and 30 vaginal deliveries and to analyze the progression of compliance with the items for each delivery until the end of the study by the RGyO at several tertiary centers in our country.

## 2. Materials and Methods

We performed a descriptive, observational study with a prospective follow-up that included the first year RG&O residents from various tertiary centers in our country who performed SW-EVD at the beginning of the training program, prior to providing medical care in the delivery clinic in which they learned the application of the CL of care for patients experiencing EVD, completing it in the first 25–30 consecutive deliveries. All residents who attended SW-EVD repeated the skills required to assist a EVD under the direct supervision of an experienced tutor at least three times, until the skills and the CL were properly learned and assimilated. There was no written evaluation recorded.

**Figure 1:**
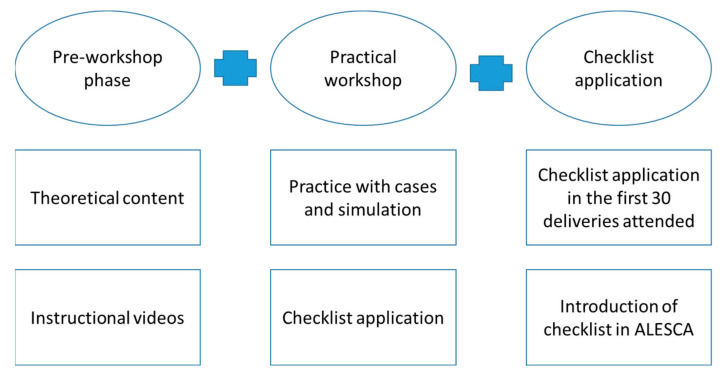
SW-EVD phases. Each of the activities to be performed by the gynecology and obstetrics residents in each phase is described.

The Hospital General Universitario Gregorio Marañón (HGUGM) was the promoter and coordinator of the study, offering SW-EVD at different national centers in which the training was conducted during September and October 2020, with follow-ups being performed from September 2020 to August 2021. The phases of SW-EVD are shown in Figure 1.

All of the theoretical content was specially designed by the HGUGM team of trainers with extensive teaching and clinical experience (including the most experienced Obstetricians and Midwives in our center). Similarly, the CL was configured by those responsible for the RGyO training, as well as the clinical managers of the emergency and delivery area of the HGUGM, taking both clinical experience and updated guidelines as reference. In Appendix A, we attach the CL of basic skills proposed in this article, used in both, SW-EVD and in their first 30 EVD assisted.

The results variable is defined as the answer “Yes”, which expresses the fulfillment of each item for each of the 30 questions proposed on the CL (questions 3 to 33). Those CLs completed during SW-EVD were not included in the analysis.

The variables analyzed for each of the participants were center of belonging, number of deliveries attended until the end of the study, isolated compliance with each item and the total number of CL items completed per delivery.

The data were collected through an online form that contained the 30 items of the CL using the ALEESCA platform, which was accessible to the participants. The possible responses to each item (Yes, No, NKNA—which stands for not knowing nor answering) were recorded for each EVD attended.

To evaluate the degree of compliance with each item constituting the CL tool, as well as the progression in its global completion until the end of the study, we only included students who had completed a minimum of 25 to 30 EVDs. Therefore, exclusion criteria included those participants who did not assist or complete at least 25 of 30 EVD and their CL and those who failed to follow up or did not attend SW-EVD.

Participant responses were included and analyzed using Microsoft Office Excel database software, version 16.42 (Microsoft, Redmond, WA, USA) during August 2021 and SPSS software (IBM, Armonk, NY, USA). Qualitative variables are described as numbers and percentages. For the quantitative data, we used the means and the 95% confidence intervals. A contrast analysis was performed using Student’s *t*-test for paired data, comparing the mean of the first five deliveries vs. the mean of the last five deliveries among the total number of participants. The cutoff point of *p* < 0.05 was considered statistically significant. There was no parallel evaluation from tutors.

All students provided oral consent to participate in the study. All women for whom delivery was assisted gave their written consent prior admission into hospital. All relevant health care providers were informed about the study, and oral consent was obtained. The participants received no compensation for participation in the study.

## 3. Results

During the data collection period between September 2020 and August 2021, a total of 7 hospital centers and 18 RGyO were included. Among all the students at the end of the study, a total of 367 CLs were completed. After applying the exclusion criteria, we finally had 2 (2/7, 28.57%) centers and 11 (11/18, 61.11%) students. Regarding the CLs, 38 (38/367, 10.35%) were finally rejected for not completing the minimum number needed, finally totaling 329 CLs.

**Figure 2:**
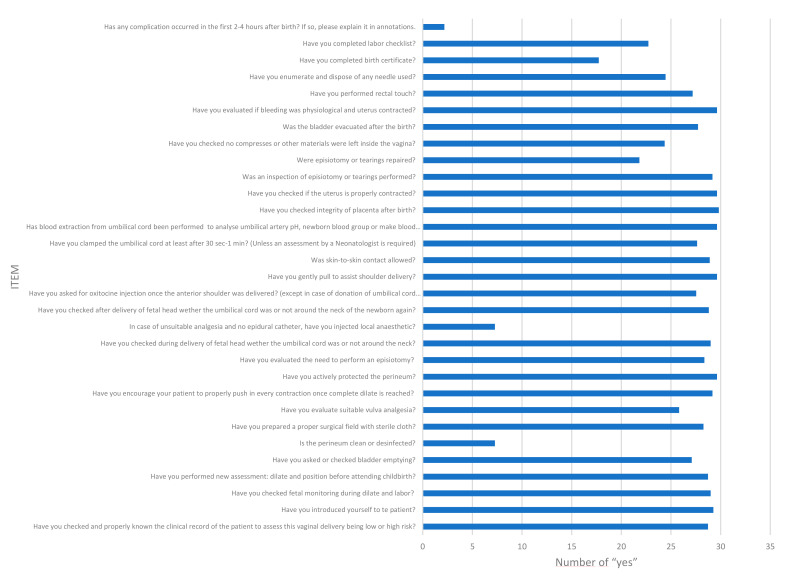
Mean number of positive responses completed on the CL for each item.

**Figure 3:**
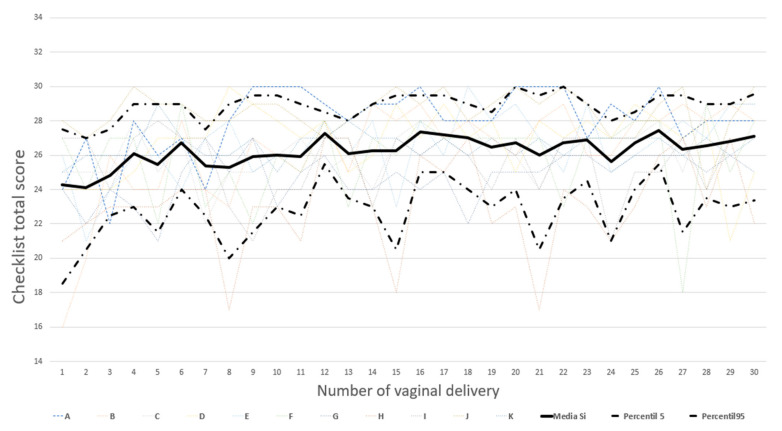
Mean CL completion for each delivery by all participants (mean of “Yes” answers for each completed CL during the first 30 deliveries). The global mean (continuous black line) of the first 30 deliveries of all participants is shown. The dashed black lines represent the 5th and 95th percentiles of mean CL completion in the first 30 births for all participants. The rest of the dashed lines of different colors reflect the individual trajectory of each participant during their care for their first 30 deliveries.

Figure 2 and Figure 3 describes the mean number of positive responses for each of the items completed on the CL. The global average of positive responses for the 30 questions contained on the CL was 25.36 points. It should be noted that question number 33, referring to whether the patient presented complications in the immediate puerperium, obtained the lowest average, with 2.18 points. Questions 8 and 15, referring to verification of disinfection of the perineum and infiltration with local anesthetic, respectively, also drew attention, obtaining scores lower than the average, both with 7.27 points. Other questions to highlight, due obtaining lower scores, were numbers 25 with 21.81 points (referring to whether the resident sutured the episiotomy or the tears produced after expulsion), 26 with 24.363 points (checking whether any compresses inserted previously into the vaginal canal were left), 30 with 24.45 points (Have you counted and disposed the used needles in the corresponding container?), 31 with 17.72 points (verification of completion of the birth certificate) and 32 with 22.72 points (verification and completion of the labor checklist). The remaining questions (22/30, 73%) obtained scores greater than 25 points.

**Figure 4:**
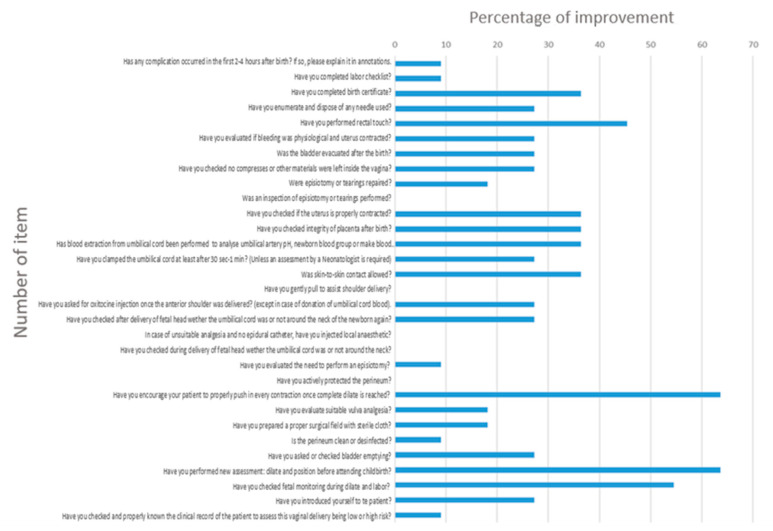
Percentage of students showing improvement and analysis of each item, after comparing the mean of “Yes” responses between the first five and last five CLs completed.

Figure 4 shows, by columns, the percentage of students who showed improvement, analyzed item by item, comparing the means between the first five and last five CL completed (corresponding to total scores reached in the first and last five EVD assisted). We observed a clear progression in 28 of 33 items. Due to their substantial improvement (more than 50% of the students), question 25 stood out (Have you sutured the episiotomy or possible tears?), as well as questions 30 (Have you enumerate and disposed of any used needle?) and 31 (Have you completed and/or verified that the birth certificate is complete?). In contrast, it is worth mentioning questions 12 (Have you actively protected the perineum), 18 (Have you gentle pull to assist shoulder delivery?), 21 (Has blood extraction from umbilical cord been performed to analyse umbilical artery pH, newborn’s blood group or make blood donation?), 22 (Have you checked integrity of the placenta after birth?) and 24 (Was an inspection of episiotomy or tearings performed?), without observing progression of the participants.

Our observations describe an upward trend in the percentage of completeness of CLs as the number of deliveries attended increased. This upward trend is supported by the statistical significance between the differences in the means of the initial births (1 to 5 mean 24.94) and those of the final ones (25 to 30, mean 26.85), with a statistically significant difference of 1.91 points (*p* < 0.05).

The low complication rate, included in the item 33, is also of note, which reads, “Has any complication occurred in the first 2–4 h after birth?”, was only completed in 2.18% of the occasions; in other words, 97.82% of deliveries being uncomplicated and of low risk.

## 4. Discussion

Again, we did not find any previous articles in the literature that presented the results of having trained 11 RGyO through SW-EVD or that evaluated them by using a CL to assist a total of 329 deliveries, each RGyO attending their first 25–30 deliveries in the usual clinical practice during the first year. However, Shumard et al. [9] and Nitsche et al. [4] worked with medical students using a CL (not described in the methodology or evaluated) throughout clinical practice in SW-EVD. Therefore, this work proposes the use of a CL for EVD care, as well as the preparation of different studies that could assess this tool for the training of future professionals.

In the aforementioned study by Shumard KM et al. [9] conducted among fourth-year medical students, only one score was greater than 26 out of 30 for the domain of advanced care of the EVD; therefore, we consider that the RGyO should at least achieve a score of 25 of 30 (83%) on the total CL to consider the successful introduction of the CL into the learning of an appropriate skill. Therefore, considering that the residents achieved more than 25 points on average on 70% of the items, showing an upward trend in the percentage of completion as the number of deliveries increased, we find improvement between the mean completion of the first five and last five childbirths attended by each RGyO, which is statistically significant (*p* < 0.05).

Regarding the evaluation of the degree of compliance of each item constituting the CL tool, we observed that some items, such as 8 (disinfection of the perineum), 15 (infiltration with local anesthetic), 25 (suture of episiotomies or tears), 26 (checking the removal of compresses inserted into the vaginal canal), 30 (checking, counting and disposal of needles used), 31 (checking completion of the birth report) and 32 (checking completion of the labor checklist) generated comprehension doubts in their completion in those cases in which it was not necessary to perform these actions (for example, in cases of regional anesthesia, complete perineum or performance of the action by another person). A solution could perhaps be to replace these statements with “Have they requested the cleaning of the perineum before attending to the delivery” or “Have they infiltrated with local anesthetic or verified the correct anesthesia of the perineal region” and clarify in writing those situations in which it was not necessary to perform sutures, a compress had not been inserted into the vaginal canal or another person had completed the labor or birth report monograph or discarded the needles used and marked “Yes” if said actions had been corroborated, even if they were not performed by the resident. In addition, it seems important to note that, although item 33, which reads, “Has any complication occurred in the first 2–4 h after birth?”, was only completed in 2.18% of the occasions, it should be recorded as a positive result since it reflects that 97.82% of deliveries being low risk and, thus, is a pertinent item. We should consider that complications for EVD include maternal and neonatal outcomes, and, sometimes, they can occur after 24 h, such as puerperal fever, delayed post-partum hemorrhage, poor wound healing and many others. Therefore, the study of these adverse outcomes could be a point of improvement for the application of CL in the future. In addition, no comparison was made about the complications rate prior to the use of CL proposed, so it may be of interest in further studies.

It is noteworthy that, in delivery number 1, 54% of the residents (6/11) obtained a total CL score greater than or equal to 25 points. This finding can be explained by the attendance of the participants to the SW-EVD in the previous weeks, as supported by a 2014 literature review, which showed an increase in the knowledge, technical, communication and work skills of the team after simulations [3,10].

As seen in Figure 4, a clear progression was observed in 28 of 33 items, with questions 25, 30 and 31 standing out due to their substantial improvement (greater than 50% of the students). This outcome could be due to an increase in confidence and acceptance of responsibility by the participants, who acquired better skills as the number of treated EVD increased and, consequently, the repetition of said CL items. In contrast, it is worth mentioning questions 12 (Have actively protected the perineum), 18 (Have you gentle pull to assist shoulder delivery?), 21 (Has blood extraction from umbilical cord been performed to analyse umbilical artery pH, newborn’s group or make blood donation?), 22 (Have you checked integrity of placenta after birth?) and 24 (was an inspection of the episiotomy or tearings performed?), in which no progression was observed on the part of the participants, perhaps because they were previously internalized basic and safety concepts, which were systematically completed by most of the participants from the first CL. We consider their removal from CL counterproductive since they are safety concepts that reinforce good clinical practice.

If we consider the initial situation, we observe that 73% of the items obtained, in isolation, average scores greater than 25 points, reinforcing the usefulness of the SW-EVD and the homogenization in clinical practice after the workshop, given that the participants internalized a significant percentage of the steps in clinical care. It is possible that the decrease in the percentage of participation in the study, especially considering those centers and their residents that did not complete at least 25 CLs, was due to rapid learning of the items of the CL, which could cause the RGyO to consider mastering the skills necessary to attend a EVD, seeing little additional benefit in continuing to complete the CL until delivery number 30.

In contrast, as seen in the graph, a significant percentage of students did not internalize the CL, as represented by the rollercoaster morphology graphs, and it would be advisable to be able to undertake teaching measures with these participants before they reached delivery number 30.

Following current indications from Spanish Society of Obstetric an Gynecology, efforts in clinic security should focus in the prevention of incidentals associated in EVD assistance [11]. Therefore, once again, we want to emphasize that the use of this tool should be implemented in the training of residents in the first year of residency since the training and qualification of the different professionals who attend the birth process are essential to reducing both maternal and fetal mortality and morbidity [7]. Although the quality of the studies is heterogeneous and with varied indicators, the evidence has shown that simulation-based teaching is effective and leads to better and more lasting results than traditional teaching, both in routine situations and in times of emergency. This benefit is seen, in part, by the increased security achieved by the participants in this type of learning, as they find themselves in a risk-free environment that allows for the student to be placed in different complex situations without harming third parties. CLs were described as a cognitive tool that allows the obstetric team assisting patients to remember critical steps needed during an emergency situation in a study about CL use in the management of obstetric hemorrhage [12], so it is understandable to consider CL to be useful in other emergencies, such as EVD assistance. In fact, as shown in the study by Vani K et al. [13], in which CL was implemented in different emergency situations in obstetrical emergencies, this tool allowed more suitable actions and management in contrast to those participants who did not use CL. In one study, the CL was found to be easy, convenient, and useful. The teams completed the CL in 2 to 3 min and showed better teamwork, communication, and decision making [2].

As a strength of the study, we consider important the large number of deliveries attended by RGyO during the 11-month period, which has made it possible to analyze the internalization of the CL as an essential part of our analysis.

In addition, we can understand that the participants in our study constitute a homogeneous sample and start from a similar knowledge base since they are all first-year residents, so we can consider the results of completing the CL to be reliable and consistent with what might be expected. according to prior knowledge.

Conversely, it could be doubted that more than 50% of the students, at the initial point, completed the CL satisfactorily (25 or more points), which could be due to the self-assessment itself. This fact could be evaluated in a future study by monitoring compliance with a CL by an experienced obstetrician tutor. We should also consider the low number of participants (11 RGyOs) participating in this study, in spite of the number of EVD analyzed.

These data could be included within the limitations of the study, together with the ability to extend the CL collection period, allowing time for the participation of more students and more centers, as well as providing plausible alternatives to those items that could give rise to subjective interpretations by the students.

Despite these limitations, our study is the first to evaluate the use of a CL by RGyO after SW-EVD, obtaining compliance of up to 78%, representing homogenization and improving clinical care for low-risk EVD.

It should be noted that, despite having obtained a significant number of completed CLs, we must be aware that up to 28.57% of the residents at 61.11% of the centers were excluded for not meeting the study criteria at the closing of the study period. This percentage of loss seems relevant to us and could be explained by, among other issues mentioned above, the difficulty in coordinating centers, as well as the attrition bias of the participants. Compliance issues could be unique to certain individuals or might reflect the need to review the entire implementation and usage system. If compliance issues are identified, the team will need to consider how best to address the issues and find solutions [14]. Regarding the large percentage of centers excluded, despite not finding studies that had the same design and that also included multiple centers, the results of this study could be due to an insufficient number of births attended by the residents in the afore mentioned centers during the stipulated study period. This idea would be supported by the average number of residents provided by each excluded center (being approximately 1) and that, moreover, in those centers, the population density is often lower, in addition to the fact that the number of deliveries at national level is decreasing, from 4.2% in the first half of 2020 to 1.3% in 2021 [15]. In addition, it is possible that the preparation of a CL composed of 30 items based on the consensus of different professionals could generate difficulties in implementation in other centers.

## 5. Conclusions

Our study proposes the use of a 30-item CL, which is progressively acquired and could be a useful tool in the training of first-year residents. In conclusion, knowing the importance of using a CL to anticipate risk situations and reduce the number of adverse perinatal outcomes, for first-year RGyO attending a SW-EVD, it seems useful to have a tool such the CL proposed in this study. During the study period, after conducting SW-EVD training in 7 centers with 18 RGyOs, we finally had the results of 11 residents and 329 CL from two hospital centers. Our results show that, in first-year residents, the CL reached more than 25 points on average in more than 70% of the items and showed an upward trend as the number of deliveries attended increased, finding a significant difference (*p* < 0.01) in the mean compliance for the first five births vs. the last five. In addition, it should be noted that the application of the CL items in the first 25 to 30 deliveries reached a compliance percentage of 73%, with a significant improvement of 1.91 points between the first and last deliveries attended, leading us to think that acquisition of the necessary skills for its completion and application is quick and effective. Such a positive result in the correct application of the items included in the CL could lead to an improvement in the RGyOs clinical skills by assisting EVD in a systematic procedure, which may reduce mistakes. However, there are few data and studies published in the literature, so it would be important to be able to perform more studies demonstrating the possible usefulness of the proposed CL, considering in optimal conditions, as is the case in our study, that the CL must be adapted to each patient and situation, according to the characteristics of the population and the clinical practice of the center.

## Data Availability

The data used to support the findings of the present study are available from the corresponding author upon request.
